# Exosomes Derived From Septic Mouse Serum Modulate Immune Responses via Exosome-Associated Cytokines

**DOI:** 10.3389/fimmu.2019.01560

**Published:** 2019-07-12

**Authors:** Kun Gao, Jingmiao Jin, Chenyang Huang, Jianhang Li, Haihua Luo, Lei Li, Yukai Huang, Yong Jiang

**Affiliations:** Guangdong Provincial Key Laboratory of Proteomics, State Key Laboratory of Organ Failure Research, Department of Pathophysiology, School of Basic Medical Sciences, Southern Medical University, Guangzhou, China

**Keywords:** sepsis, inflammation, exosome, T cell differentiation, cytokine

## Abstract

Sepsis is a life-threatening condition caused by an immune response triggered by infection, and highly elevated cytokine/chemokine levels in the blood play crucial roles in the progression of sepsis. Serum exosomes are nanovesicles that have multiple biological functions, playing roles in antigen presentation, intercellular signal communication, inflammatory response and immune surveillance. However, the biological functions and related molecular bases remain to be elucidated. In this study, we investigated the profiles of cytokines/chemokines harbored in the exosomes of septic mice and explored the mechanisms of immunomodulation on T cells treated with exosomes harvested from septic mice. Blood cytokines/chemokines existed in both the soluble form and in the insoluble exosomal form; the profiles of the cytokines/chemokines in these two forms displayed different dynamics in the blood of mice challenged with LPS. Exosomes from septic mice induced the differentiation of Th1/Th2 cells, which was blocked by specific antibodies targeting IL-12 and IL-4. In addition, these exosomes significantly augmented the proliferation and migration of T lymphocytes. Furthermore, preadministration of exosomes by intravenous injection restrained the inflammatory response, attenuated lung and liver tissue damage, and prolonged the survival of cecal ligation and puncture (CLP) mice. Our results indicate that exosomes enriched with cytokines/chemokines play critical roles in T cell differentiation, proliferation and chemotaxis during the sepsis process and have a protective effect on cecal ligation and puncture (CLP) mice. Thus, these findings not only strengthen our understanding of the role of sepsis via exosomes but also provide potential targets for therapeutic applications.

## Introduction

Sepsis, the primary cause of death in infection patients, is a strong immune response to a complicated infection. With the development of sepsis, proinflammatory cytokine levels in the blood are significantly elevated (1–4), and Bone (5) noted that “cytokine storm” mortality remains elevated for a long period after a septic episode is resolved. During sepsis, high levels of proinflammatory and anti-inflammatory cytokines are circulating, and immune responses, including the activation, proliferation and differentiation of T cells, play critical roles in the regulation of sepsis (5, 6). CD4^+^ Th cells are the most important lymphocyte subset influencing innate and adaptive immune cells via cytokines and cell-to-cell interactions (7). Th1 cells are induced by IL-12 and IFN-γ in response to viral, bacterial and protozoan intracellular infections, and Th2 cells activated by IL-4 are important for the clearance of helminth infections (8, 9).

Exosomes, extracellular vesicles 30–120 nm in diameter, are released by various cells and have various physiological functions, as they transport proteins, messenger RNAs (mRNAs) and non-coding RNAs (ncRNAs); facilitate intercellular communication; and elicit immune responses (10, 11).

Diverse classes of biomolecules are packaged, coexpressed, and secreted from cells into bodily fluids to interact with other cells (12, 13), and biomolecules traveling a long distance are transported via a novel pathway. The production of exosomes can be spontaneously secreted depending on the cell type (14, 15) or induced by various stimuli (16). The biochemical composition of exosomes is variable depending on their cell origin (17), and exosomes have been shown to play key roles in regulating immune responses. The major histocompatibility complex (MHC)-peptide complexes and antigens carried in exosomes are crucial for initiating and amplifying the immune response (18, 19), and determining whether circulating cytokines are released in a controlled manner as a compensatory mechanism in response to a stress signal is important. Konadu et al. (20) reported that HIV-infected and viremic individuals exhibited elevated levels of plasma cytokines, and most cytokines were colocalized with exosomes derived from mouse serum. Twenty-one cytokines and chemokines are markedly enriched in HIV-positive exosomes to negatively control and induce the expression of CD38 on CD4^+^/CD8^+^ T cells. The stimulation of RAW 264.7 cells by lipopolysaccharide (LPS) leads to increased exosomal chemokine and RNA levels and is thus involved in the regulation of inflammation (21). In this study, we isolated exosomes from the sera of septic mice, quantified their cytokine levels and assessed their immunomodulatory effect on lymphocytes to confirm our hypothesis that cytokines can be transferred among cells in septic mouse serum via an exosome secretion mechanism.

## Methods

### Antibodies and Reagents

Mouse pan T cell isolation kit II (Cat.130-095-130), MACS BSA stock solution (Cat.130-091-376), and autoMACS BSA rinsing solution (Cat.130-091-222) were purchased from Miltenyi Biotec (Bergisch Gladbach, Germany). Mouse Th1/Th2/Th17 Phenotyping Kit (Cat.560758), Leukocyte Activation Cocktail with BD GolgiPlug™ (Cat.550583), Lysing Buffer (Cat.555899), PerCP-Cy™5.5 Rat Anti-Mouse CD4 (Cat.550954), FITC Rat Anti-Mouse IFN-γ (Cat.562019), APC Rat Anti-Mouse IL-4 (Cat.562045), PE Rat Anti-Mouse IL-17A (Cat.561020), FITC Rat Anti-Mouse CD4 (Cat.553046), APC Rat Anti-Mouse CD25 (Cat.557192) were purchased from BD Pharmingen (Franklin Lakes, NJ, USA). Antibodies against mouse CD9 (Cat.ab223052), CD63 (Cat.ab216130), or CD81 (Cat.ab109201) and phorbol 12-myristate 13-acetate (PMA) (Cat.ab120297) were purchased from Abcam (Cambridge, MA, USA). Antibodies against mouse IL-12 (Cat.AF-419-SP) and IL-4 (Cat.MAB404) were from R&D Systems (Minneapolis, MN, USA). Concanavalin A (ConA) (Cat.C2272) and lipopolysaccharides (LPS) from *Escherichia coli* O111:B4 (Cat.L2630) were from Sigma-Aldrich (St. Louis, MO) and CellTrace™ CFSE Cell Proliferation Kit (Cat.C34570) was from Invitrogen (OR, USA). Transwell® polycarbonate membrane cell culture inserts (Cat. CLS3422) was provided by Corning (Corning, NY, USA). Fetal bovine serum (FBS) (Cat.10099141) and Dulbecco's Modified Eagle Medium (DMEM) (Cat.C11995500BT) were from Thermo Fisher (Cambridge, MA, USA). Recombinant Murine Exodus-2 (CCL21) (Cat. 250-13) was from PeproTech (Rocky Hill, NJ, USA). The MILLIPLEX MAP Mouse Cytokine/Chemokine Magnetic Bead Panel (Cat. MCYTOMAG-70K) was from Millipore (Burlington, MA, USA). Polymyxin B (PMB) (Cat.1405-20-5) was purchased from Calbiochem (San Diego, CA, USA).

### Mice and Sepsis Modeling

C57BL/6 mice were purchased from the experimental animal center of Southern Medical University (Guangzhou, China), and TLR4 knockout C57BL/6 mice were kindly provided by Dr. T.R. Billiar (Department of Surgery, University of Pittsburgh, USA). Male mice were housed in a specific pathogen-free facility. Male C57BL/6 mice aged 8–9 weeks (weighing 21.6 ± 0.8 g) were intraperitoneally injected with LPS (10 mg/kg) in sterile phosphate-buffered saline (PBS) to reproduce the sepsis model. All animal experiments were approved by the Animal Welfare and Ethics Committee of Southern Medical University, Guangzhou, China.

### Isolation of Exosomes From Septic Mouse Serum

According to a previously described method (22), exosomes were isolated using a modified differential ultracentrifugation protocol (Beckman Coulter). The first step, centrifugation at 2,000 × g for 20 min at 4°C, was designed to eliminate large cell fragments or debris. The supernatant was collected and centrifuged at 12,000 × g at 4°C for 45 min to remove small cellular debris. Then, the supernatant was transferred to a new tube and ultracentrifuged at 120,000 × g for 120 min at 4°C to pellet the small vesicles. The supernatant was discarded, and the pellet was resuspended in a large volume of PBS and then filtered with a 0.22 μm filter to eliminate potential contaminants. After ultracentrifugation at 120,000 × g for 120 min at 4°C, the pellets were resuspended in PBS, and protein quantitation was performed using a bicinchoninic acid (BCA) assay kit (Pierce, USA). Exosomes extracted from the blood of mice challenged with LPS for different amounts of time (0, 2, 12, 24, and 48 h) were defined as Exo-0, Exo-2, Exo-12, Exo-24, and Exo-48, respectively.

### Preparation of Exosome-Free Medium

Exosomes were depleted from FBS-containing medium according to the protocol described by Thery C (23). In brief, 10 ml of complete DMEM supplemented with 20% FBS was prepared by centrifugation at 100,000 × g overnight at 4°C. The supernatant was sterilized with a 0.22 μm filter unit driven by a vacuum system.

### Exosome Characterization

Exosomes were purified from the sera of septic mice, and transmission electron microscopy (TEM) and nanoparticle tracking analysis (NTA) were used to confirm the quality of the exosome preparations. Western blot analyses were performed with specific antibodies (Abs) against exosome biomarker proteins, e.g., CD9, CD63, and CD81. Serum samples from mice challenged with LPS for different amounts of time and exosomes derived from the sera were analyzed with flexible multi-analyte profiling (xMAP) technology using a Luminex-200 system for cytokine quantitation (1, 24, 25).

### Isolation of Splenic Lymphocytes and Purification of T Cells

The spleens obtained from C57BL/6 mice were ground on 200-mesh sieves. The suspensions were centrifuged at 300 × g for 8 min, and the pellets were mixed with 10 × lysis buffer and incubated at 37°C for 5 min to remove the red blood cells. The resuspended mixture was centrifuged at 800 × g for 30 min in lymphocyte isolating solution, and the lymphocyte layer was collected. T cells were purified using the Pan T cell isolation kit according to the manufacturer's instructions. T lymphocyte purity was determined using the anti-CD3-APC mAb by fluorescence activated cell sorting (FACS) analysis.

### FACS Analysis of T Cells

To determine the biological effects of exosomes from septic mice on T cell differentiation *in vitro*, we incubated splenic lymphocytes with ConA (5 μg/ml) and exosomes (10 μg/ml) purified from mice with or without sepsis for 72 h. Fluorescent Abs specifically targeting mouse CD4, IFN-γ, IL-4, and IL-17 were used for the flow cytometry analysis according to the manufacturer's instructions.

To validate the results obtained using septic mouse exosomes, we purified splenic lymphocyte T cells by the negative depletion of magnetically labeled cells. Using beads coated with anti-CD3 and anti-CD28 to stimulate T cells is a physiologically relevant approach to mimic stimulation by antigen-presenting cells (26). Thus, we incubated the cells with anti-CD3/CD28-coated beads and purified exosomes (10 μg/ml) for 72 h for flow cytometry analysis.

To further confirm the biological effects of the exosomes harvested from septic mice, we performed a blocking assay using exosomes preincubated with neutralizing Abs specifically targeting mouse IL-12 (1 μg/ml) or IL-4 (1 μg/ml) at 37°C for 1 h to block the action of IL-12 or IL-4.

### Cell Proliferation Assays

Cell proliferation was measured by flow cytometry analysis. Splenic lymphocytes were stained with carboxyfluorescein diacetate succinimidyl ester (CFSE) at a final concentration of 5 μmol/L for 10 min at 37°C. The CFSE-stained cells (5 ×10^5^) were incubated with ConA (5 μg/ml) and the purified exosomes (10 μg/ml) for 5 days. The cells were then collected and analyzed by flow cytometry.

### Chemotaxis Assays

To determine the effect of exosomes on lymphocyte migration, we performed a transwell assay under two different conditions. In the first condition, the upper chamber was seeded with lymphocytes, and the bottom chamber was seeded with exosomes; the chambers were then incubated at 37°C for 8 h. In the second condition, after pretreatment with 10 μg/ml exosomes for 24 h, lymphocytes were seeded in the upper chamber, and 10% FBS was added to the bottom chamber; the chambers were then incubated at 37°C for 8 h. Chemoattracted lymphocytes in lower well were resuspended in 1 ml medium and the absolute cell number was counted by Hemocytometer.

### *In vivo* Studies

C57BL/6 mice were injected with exosomes (100 μg/mouse) via their tail vein 3 times on days 1, 3, and 5. The cecal ligation and puncture (CLP) mouse model was established on day 7 (27), and the survival times of the mice were observed. To clarify the effect of exosomes on the levels of TNF-α and IL-10, mouse blood was collected at 8 h after CLP modeling for analysis with xMAP technology. Lung and liver tissue injuries in septic mice were evaluated by hematoxylin eosin (HE) staining. In brief, the lung or liver tissues were fixed with 10% formaldehyde and embedded in paraffin for the preparation of tissue sections (4 μm). The tissue slides were stained with HE and observed under an Axio Imager Z2 microscope (Carl Zeiss, Oberkochen, Germany) for pathological evaluation.

### Air Pouch Animal Model

Mouse air pouches were prepared as previously described (28). In brief, mice were anesthetized and then injected with 5 ml of sterile air under their dorsal skin. On day 3, the resultant space was again injected with 3 ml of sterile air. On day 5, exosomes from mice challenged with or without LPS or CCL21 were injected into the dorsal air pouches. The air pouches were washed with PBS after a fixed migration time of 12 h, and the lavage fluids were collected for lymphocyte counting.

### Data Analysis

Data are presented as the mean ± standard deviation (SD). One-way analysis of variance (ANOVA) in combination with the least squares difference (L.S.D) test was utilized to analyze differences among multiple groups using Statistical Package for the Social Science software (SPSS, v22.0). Paired *t*-tests were utilized to analyze differences between two groups. The Kaplan-Meier method was used to evaluate survival curves. *P* <0.05 were considered statistically significant.

## Results

### Characterization of Exosomes Derived From Septic Mouse Serum

**Figure 1:**
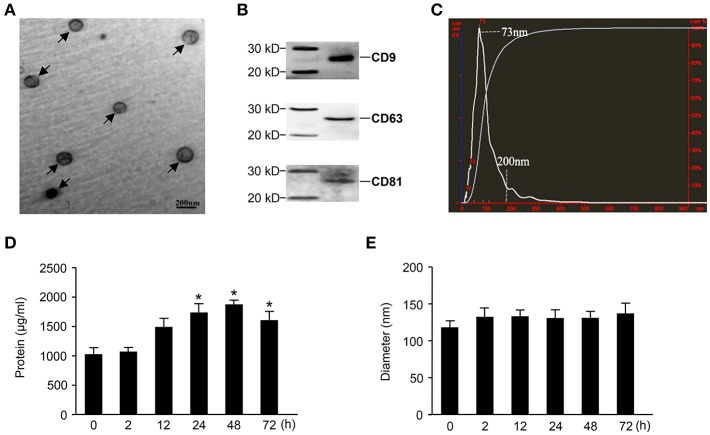
Characterization of exosomes derived from septic mouse serum. **(A)** Electron microscope (EM) analysis of exosomes. The circular particles representing exosomes are indicated by arrows. The scale bar represents a length of 200 nm. **(B)** Exosomes containing 20 μg of proteins were used to detect exosome-specific proteins (CD9, CD63, and CD81) by Western blot. **(C)** The size distribution of exosomes was determined by nanoparticle tracking analysis (NTA). **(D)** Dynamic change in exosome protein concentrations. The exosomes were separated from 200 μl of serum harvested from septic mice at different time points for protein quantitation. **(E)** The sequential detection of exosomes derived from septic mouse serum. The particle sizes of exosomes were analyzed by the NTA technique. Data are shown as the mean ± SD from three independent experiments (*n* = 3) and analyzed by one-way ANOVA. ^*^P < 0.05, compared with the 0 h as the control.

Exosomes were purified from the sera of mice in which sepsis was induced by the intraperitoneal injection of 15 mg/kg LPS. Then, an electronic microscope (EM) was used to measure the diameters of the purified exosomes, revealing that all types of exosomes were displayed as round vesicles with an approximate diameter of 50–150 nm (Figure 1A). Exosome-associated proteins, including CD9, CD63, and CD81, were detected by Western blot analysis (Figure 1B). To further verify the exact diameter of the exosomes, the exosomes derived from C57BL/6 mouse sera were subjected to Nanosight NTA, revealing an approximate diameter of 91 nm (Figure 1C). To clarify the effects of LPS challenge on the protein contents and diameters of exosomes, we compared the protein concentration and diameters of exosomes derived from the sera of mice challenged with LPS for different amounts of time. Interestingly, LPS treatment significantly increased the protein content of serum exosomes (Figure 1D). However, we failed to detect any significant difference in the diameters of exosomes collected at different time points (Figure 1E).

### Expression Profiles of Inflammatory Cytokines, Chemokines, and Growth Factors in Exosomes Purified From Septic Mice

Both cytokines and chemokines play important roles in the progression of sepsis. Thus, we analyzed the expression profiles of inflammatory cytokines, chemokines, and growth factors in exosomes derived from the sera of mice challenged with LPS for different amounts of time.

All 18 cytokines, chemokines, and growth factors, including interleukin-1β (IL-1β), IL-2, IL-4, IL-5, IL-6, IL-10, IL-12, IL-15, IL-17, tumor necrosis factor-α (TNF-α), interferon-γ (IFN-γ), chemokine (C-C motif) ligand 2 (CCL2), CCL3, CCL5, chemokine (C-X-C motif) ligand 9 (CXCL9), CXCL10, granulocyte-macrophage colony stimulating factor (GM-CSF), and vascular endothelial growth factor (VEGF), were detected in the exosomes isolated from septic mice.

**Figure 2:**
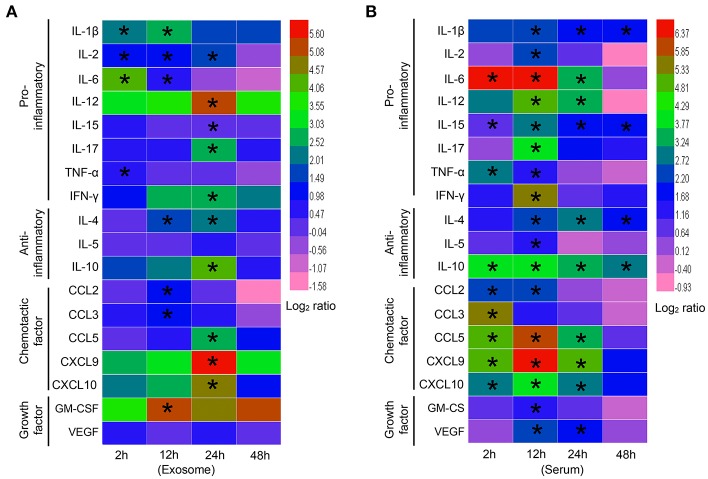
Expression profiles of exosomal cytokines in septic mouse serum. **(A)** Cytokine profiles of exosomes harvested from septic mice. Exosomes were extracted from the blood of septic mice at different time points and lysed by RIPA lysis buffer. Proinflammatory cytokines (IL-1β, IL-2, IL-6, IL-12, IL-15, IL-17, TNF-α, and IFN-γ), anti-inflammatory cytokines (IL-4, IL-5, and IL-10), chemokines (CCL2, CCL3, CCL5, CXCL9, and CXCL10) and growth factors (GM-CSF and VEGF) were detected by flexible multianalyte profiling (xMAP) technology. The results at each time point were divided by that of the Exo-0 group and log_2_ transformed to show the log-fold change after stimulation. **(B)** Cytokine profiles of septic mouse serum. Exosomes were depleted from the sera of septic mice for the analysis of cytokine expression. Data are shown as the mean ± SD (*n* = 3) and analyzed by ANOVA. ^*^P < 0.05 vs. the Exo-0 group.

For the proinflammatory cytokines, the levels of IL-1β, IL-2, IL-6, and TNF-α were elevated in the early sepsis phase (2 h), but most other proinflammatory cytokines, including IL-12, IL-15, IL-17, and IFN-γ, showed significant elevation in the late sepsis phase (24–48 h) (Figure 2A). For the anti-inflammatory cytokines, IL-4 and IL-10 were expressed at high levels in the late phase, whereas IL-5 expression was not significantly altered at any of the time points studied (Figure 2A). For the chemokines, CCL2 and CCL3 levels were elevated in the middle sepsis phase (12 h), while CCL5, CXCL9, and CXCL10 levels were significantly increased in the late phase. For the growth factors, GM-CSF reached a high level at 12 h, whereas VEGF expression was not significantly altered during any of the sepsis phases (Figure 2A).

Upon comparing the exosome results, we indeed found significant differences in the cytokines harvested from the exosome-depleted sera of septic mice. More factors, especially chemokines, were involved in early phase responses via the free form than via the exosome-associated form (Figure 2B).

Together, these results suggest that cytokines, chemokines, and growth factors in the blood exist in two different states, a soluble form and an insoluble form associated with exosomes. Importantly, exosome-associated cytokines might perform specific biological functions via different mechanisms.

### Exosomes From Septic Mice Augmented the Differentiation of Th Cells

To determine whether the cytokines associated with exosomes harvested from septic mice were biologically active, we tested the effect of the sepsis-associated exosomes on T cell differentiation *in vitro* by incubating the exosomes with ConA-activated splenic lymphocytes.

**Figure 3:**
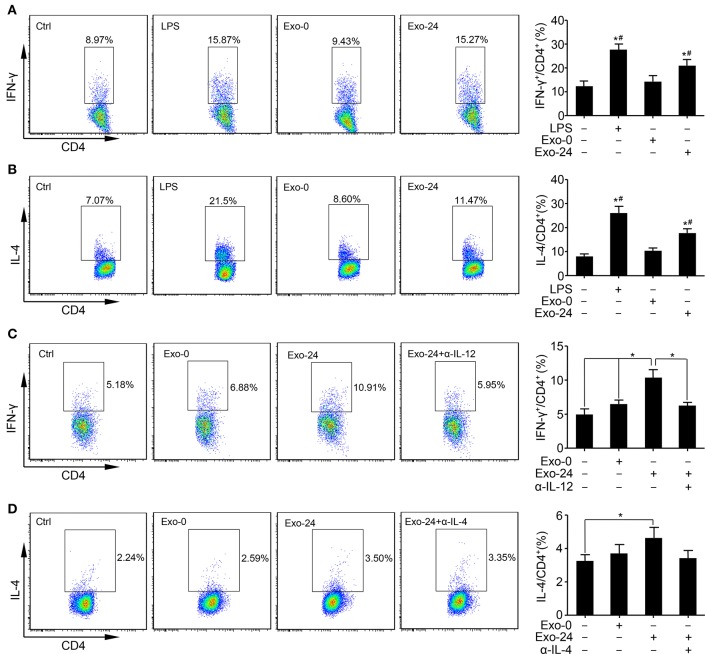
Exosomes from septic mice augmented Th1 and Th2 differentiation. **(A)** The Th1 response was augmented by exosomes harvested from septic mouse serum. Lymphocytes were purified from the spleens of C57BL/6 mice and incubated with LPS (1 μg/ml) or exosomes (Exo-0 or Exo-24, 10 μg/ml) for 72 h. Flow cytometry analysis was performed to determine the percentage of Th1 cells by quantitating the ratio of IFN-γ-expressing cells to CD4^+^ T cells. Lymphocytes stimulated with LPS for 72 h (LPS group) were used as a positive control. Data are presented as the mean ± SD (*n* = 4), ^*^P < 0.05 vs. the control group; ^#^*P* < 0.05 vs. the Exo-0 group. **(B)** The Th2 response was augmented by exosomes harvested from septic mouse serum. Splenic lymphocytes were acquired as described above. After incubation with LPS, Exo-0 or Exo-24 for 72 h, the percentage of Th2 cells was quantitated by the ratio of IL-4-expressing cells to CD4^+^ T cells by flow cytometry. Lymphocytes stimulated with LPS for 72 h (LPS group) were used as a positive control. Data are presented as the mean ± SD (*n* = 4), ^*^P < 0.05 vs. the control group; ^#^*P* < 0.05 vs. the Exo-0 group. **(C)** The blocking effect of a neutralizing antibody against IL-12 on the Th1 response augmented by exosomes harvested from septic mice. Purified T cells from splenic lymphocytes of C57BL/6 mice were incubated with exosomes (Exo-0 or Exo-24, 10 μg/ml) for 72 h. For the blocking assay, exosomes from the sera of septic mice (Exo-24) were preincubated with an IL-12 neutralizing antibody (1 μg/ml) at 37°C for 1 h (Exo-24+α-IL-12 group) to test its inhibitory effect on the Th1 response. The percentage of Th1 cells was determined by the method described above. Data are presented as the mean ± SD (*n* = 4), ^*^P < 0.05, the Exo-24 group vs. the control, Exo-0 and Exo-24+α-IL-12 groups. **(D)** The blocking effect of a neutralizing antibody against IL-4 on the Th2 response augmented by exosomes harvested from septic mice. T cells were purified and incubated as described above. For the blocking assay, exosomes from the sera of septic mice (Exo-24) were preincubated with an IL-4 neutralizing antibody (1 μg/ml) at 37°C for 1 h (Exo-24+α-IL-4 group) to test its inhibitory effect on the Th2 response. The percentage of Th2 cells was quantitated as described above. Data are presented as the mean ± SD (*n* = 4), ^*^P < 0.05 vs. the control group.

FACS analysis revealed that the percentages of Th1 (Figure 3A) and Th2 (Figure 3B) cells among the total splenic T cells were increased by treatment with exosomes harvested from septic mice for 24 h (Exo-24) compared to those in the controls. We also assessed the effects of the exosomes on the differentiation of Th17 cells but failed to obtain any positive results (Supplementary Figure 1).

To test whether the biological effects on Th1/2 differentiation were TLR4-dependent, we incubated the exosomes with splenic lymphocytes derived from TLR4 knockout mice. Interestingly, compared with that in the controls, vigorous Th1/Th2 cell differentiation was observed in the Exo-24 group (Supplementary Figures 2A,B), indicating that the biological activity of the exosomes harvested from septic mice was not attained via the ability of the LPS receptor TLR4 to transduce signals.

It is well-established that mouse splenic lymphocytes consist of T cells, B cells, macrophages, and dendritic cells. Thus, we wanted to determine whether exosome-mediated T cell differentiation was a direct result of T cell actions or an indirect result of other immune cell actions. Pure T cells were obtained with a purification rate of 96% by using a Pan T cell isolation kit (Supplementary Figure 3). Consistent with the splenic lymphocyte results, exosomes harvested from septic mice directly augmented the Th differentiation of purified T cells, especially altering the Th1 response.

Previous studies demonstrated that IL-12 and IL-4 are involved in the differentiation of naïve T cells into Th1 and Th2 cells, respectively. Thus, we performed a blocking assay with neutralizing Abs and found that Abs specifically targeting mouse IL-12 (Figure 3C) and IL-4 (Figure 3D) remarkedly abrogated the activity of Th cell differentiation induced by exosomes harvested from septic mice.

### Exosomes From Septic Mice Enhanced Lymphocyte Cells Proliferation and Migration

**Figure 4:**
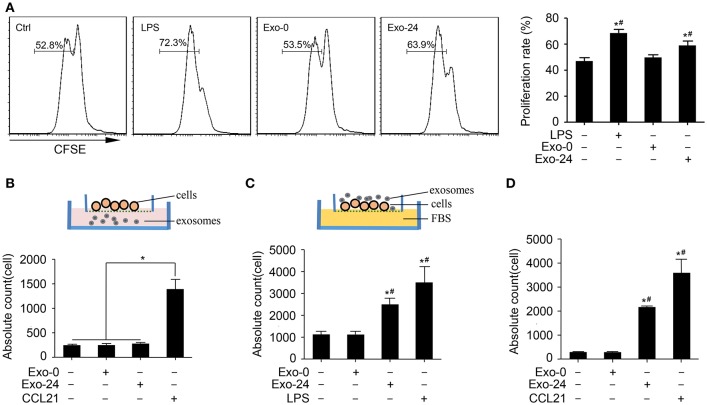
Exosomes from septic mice augmented the proliferation and migration of lymphocytes. **(A)** Lymphocyte proliferation was enhanced by exosomes harvested from septic mice. Splenic lymphocytes were stained with CFSE and incubated with Exo-0 or Exo-24 for 72 h. Proliferation was quantitated as the percentage of CFSE_low_ cells by FACS. Lymphocytes challenged with LPS for 72 h (LPS group) were used as the positive control. Data are presented as the mean ± SD (*n* = 4). ^*^P < 0.05, vs. the control group; ^#^*P* < 0.05, vs. the Exo-0 group. **(B)** Exosomes from septic mice failed to directly induce lymphocyte migration. Splenic lymphocytes were seeded on the bottom of the upper transwell unit, and exosomes (Exo-0 or Exo-24, 10 μg/ml) or CCL21 (1 μg/ml) was then added to the lower chamber to perform the transwell migration assay. The migration lymphocytes were counted after 8 h. Data are presented as the mean ± SD (*n* = 3). ^*^P < 0.05, CCL21 group vs. the control, Exo-0 and Exo-24 groups. **(C)** Preincubation with exosomes harvested from septic mice enhanced lymphocyte migration. After preincubation with exosomes (Exo-0 or Exo-24, 10 μg/ml) or LPS (1 μg/ml) for 24 h, lymphocytes were seeded in the upper unit, and 10% FBS was added to the bottom unit as the chemoattractant. The lymphocytes chemoattracted to the lower unit were counted after 8 h. Data are presented as the mean ± SD (*n* = 3). ^*^P < 0.05, vs. the control group; ^#^*P* < 0.05, vs. the Exo-0 group. **(D)** Exosomes from septic mice augmented the chemotaxis of lymphocytes *in vivo*. An air pouch mouse model was reproduced, and exosomes (Exo-0 or Exo-24, 10 μg/ml) or CCL21 (1 μg/ml) were injected into the dorsal air pouches of the mice. The chemoattracted lymphocytes were counted after 12 h. Data are presented as the mean ± SD (*n* = 3). ^*^P < 0.05, vs. the control group; ^#^*P* < 0.05, vs. the Exo-0 group.

Based on the finding that the levels of growth factors and chemokines were increased in the exosomes harvested from septic mice, we attempted to clarify whether these exosomes modulate the proliferation and migration of lymphocytes. The CFSE assay results showed that treatment with Exo-24 but not Exo-0 had significant effects on the proliferation of lymphocytes activated by ConA (Figure 4A). Similarly, we tried to determine whether the proliferative effects of lymphocytes induced by the exosomes were TLR4-dependent, revealing that treatment with Exo-24 but not Exo-0 had significant effects on the proliferation of splenic lymphocytes derived from TLR4 knockout mice (Supplementary Figure 4A).

To rule out the possibility that the exosome-triggered responses arose from endotoxin contamination in the exosome preparations, we used polymyxin B (PMB) to neutralize LPS and found that PMB failed to eliminate the proliferative effect of T lymphocytes induced by exosomes harvested from septic mice (Supplementary Figure 4B).

Next, we investigated whether the exosomes harvested from septic mice could augment lymphocyte migration. While the exosomes from septic mice (Exo-24) failed to chemoattract lymphocytes, CCL21 had chemoattractant activity on splenic lymphocytes seeded on the bottom of the upper transwell unit (Figure 4B). However, preincubation of lymphocytes with exosomes harvested from septic mice (Exo-24) indeed enhanced the migration activity of lymphocytes to FBS in the bottom transwell unit (Figure 4C).

Finally, we performed an *in vivo* assay by reproducing the mouse air pouch model, which was followed by injection of exosomes harvested from septic mice (Exo-24) or CCL21 into the dorsal air pouches of the mice. Exo-24 significantly increased the absolute counts of chemoattractant lymphocytes (Figure 4D).

### Pretreatment With Exosomes Harvested From Septic Mice Reduced the Inflammatory Response and Tissue Injury in CLP Septic Mice

**Figure 5:**
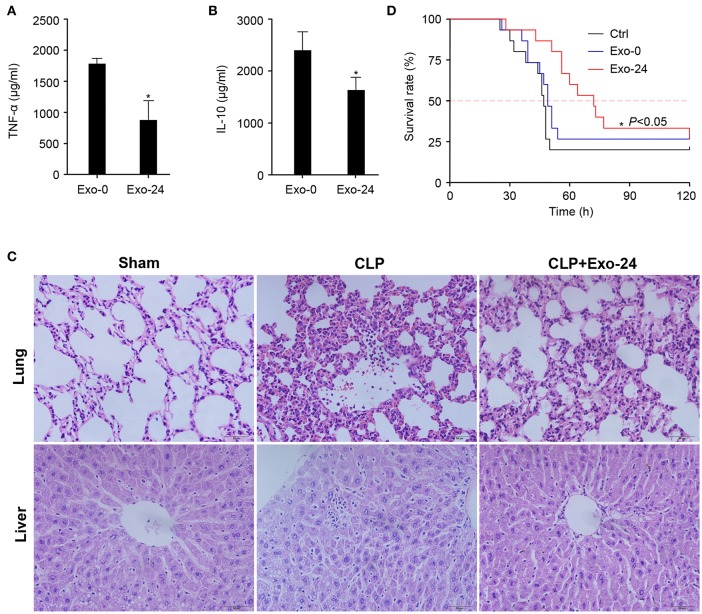
Pretreatment with exosomes from septic mice reduced the inflammatory response and tissue injury in CLP model mice. **(A)** Inhibitory effect of exosomes from septic mice on TNF-α production. Exosomes (Exo-0 or Exo-24, 100 μg/mouse) were administered to recipient mice on days 1/3/5 via tail vein injection (TVI). The CLP model was established on day 7, and sera from the mice were collected at 8 h after CLP modeling for the quantitation of TNF-α by xMAP technology. Data are shown as the mean ± SD. ^*^P < 0.05 vs. the Exo-0 group. **(B)** Exosomes from septic mice reduced the level of IL-10. After CLP treatment for 8 h, IL-10 in the serum was quantitated by xMAP technology. Data are shown as the mean ± SD. ^*^P < 0.05 vs. the Exo-0 group. **(C)** Exosomes from septic mice restrained lung and liver tissue injury in septic mice. HE staining was performed to detect the morphological changes in lung and liver tissues harvested from CLP mice either preinjected with exosomes from septic mice 3 times as described above or not. **(D)** Exosomes from septic mice significantly prolonged the survival times of CLP mice. Exosomes (Exo-0 or Exo-24, 100 μg/mouse) were intravenously injected into mice, and the CLP model was established as described above. Then, their survival times were observed, and the Kaplan-Meier method was used to evaluate survival curves. *P* < 0.05 was considered statistically significant.

Our above findings demonstrated that cytokines, chemokines, and growth factors were selectively but not randomly packaged in the exosomes of septic mice (Figure 2), which modulated the systemic inflammatory response via initiating the differentiation, proliferation and chemotaxis of T cell subsets (Figures 3, 4). To test their biological activity *in vivo*, exosomes harvested from septic mice were administered to recipient mice 3 times via tail vein injection (TVI). The CLP model was established on day 7, and the mouse sera were collected at 8 h after CLP modeling for the quantitation of cytokines. The serum levels of both the representative proinflammatory cytokine TNF-α (Figure 5A) and the anti-inflammatory factor IL-10 (Figure 5B) were decreased at 8 h after CLP modeling in mice upon the preinjection of Exo-24, but the decreased amplitude of TNF-α was greater than that of IL-10.

Twelve hours after CLP modeling, HE staining was performed to detect morphological changes in the lung and liver tissues of mice either preinjected with exosomes harvested from septic mice or not. Pathological examinations showed that the exosomes from septic mice restrained the tissue injuries in the lungs and livers of septic mice (Figure 5C).

We further performed a Kaplan-Meier survival analysis on CLP mice and intriguingly found that the preinjection of exosomes from septic mice significantly prolonged the survival times of CLP mice (Figure 5D). These results indicated that treatment with exosomes from septic mice could inhibit the development of sepsis in mice.

## Discussion

Sepsis is a lethal disease, and its progression is associated with high serum levels of cytokines, including inflammatory cytokines, chemokines, and growth factors (1, 3), which play crucial roles in the development of sepsis (29). Recent studies demonstrate that in addition to existing in the soluble form in serum, cytokines also exist in exosomes. Konadu et al. (20) reported that exosomes purified from the plasma of HIV-seropositive individuals were actively and selectively enriched for most of the screened cytokines and chemokines. Due to the stability of exosomes, the half-lives of the cytokines and chemokines within exosomes are increased (30, 31). In the present study, we provide evidence that most cytokines/chemokines not only exist in the free form but are also packaged into exosomes in the sera of mice with sepsis. Here, we showed that the protein contents of exosomes harvested from the sera of septic mice increased with disease progression and reached a maximum level at 48 h after LPS challenge (Figure 1D). But we failed to detect any significant difference of the number of exosomes from septic mice in comparison with the control (Supplementary Figure 5). The proteins in exosomes reportedly vary among different cell types (32, 33), and in the progression of sepsis, inflammatory cytokines/chemokines are released by various types of immune cells, such as T cells, B cells and macrophages. Cytokines/chemokines in both the soluble and exosomal forms reflect the complicated integration of the bioactivities of all immune cells. However, the two forms of serum cytokines/chemokines displayed different dynamics during the development of sepsis. Most of the soluble cytokines/chemokines, including TNF-α, IFN-γ, IL-2, IL-6, IL-12, IL-15, IL-17, IL-10, CCL5, CXCL9, and CXCL10, in the sera of septic mice showed increased levels, which peaked at 2–12 h, whereas most of the cytokines/chemokines in the exosomes showed delayed peak expression at 12–24 h after sepsis modeling. Growth factors, such as VEGF, also showed varying dynamics between the soluble and exosomal forms in the sera of septic mice. Together, these results suggest that most cytokines/chemokines exist in both the soluble form and the exosome form but display different dynamics during the development of sepsis.

Exosomes act as a functional unit to facilitate extracellular signal communication among cells *in vivo*. Previous studies demonstrated that exosomes derived from serum play crucial roles in carrying and presenting functional MHC-peptide complexes to modulate T cell responses (34). The components of exosomes have been shown to include peptide-bound MHC class I and II molecules, T cell stimulatory molecules (B7.2, ICAM-1) and other immune molecules (35, 36). Other studies have revealed that exosomes, including the 19-kDa lipoprotein, induce an immune cell response (23, 37).

In this study, we incubated exosomes derived from septic mice with lymphocytes and found that the exosomes enhanced Th1/Th2 cell differentiation, especially influencing the Th1 response (Figures 3A,B), which is consistent with the findings of several studies on other disease models (19, 38, 39).

To investigate whether exosomes directly enhanced T cell differentiation via cytokines *in vitro*, we purified T cells from mouse splenic lymphocytes and incubated them with exosomes from septic mice. In line with the splenic lymphocyte results, exosomes from septic mice directly augmented the Th differentiation of purified T cells (Figures 3C,D). We performed a blocking assay with neutralizing Abs specifically targeting mouse IL-12 (Figure 3C) or IL-4 (Figure 3D), revealing that the differentiation of T cells was promoted by the IL-12 and IL-4 cytokines, which were associated with exosomes harvested from septic mice. Consistent with these results, Yu et al. showed that mTGF-β1-Exo induced a vigorous Treg cell response (40). We also incubated the exosomes with purified T lymphocytes derived from TLR4 knockout mice and found that the exosomes from septic mice still enhanced Th1/Th2 cell differentiation (Supplementary Figure 2), indicating that the biological effects on Th1/2 differentiation were TLR4-independent. Together, these data implied that the exosomes from septic mice (Exo-24) had a potent capability to enhance Th1/2 cell differentiation via the actions of IL-12 and IL-4 cytokines.

Release of exosomes represents an immediate cell communication mechanism that acts in three different manners: transfer of an expression pattern to other cells, dissemination of the membrane-bound mediators within the liquid phase and rapid rearrangement of the cell surface (41). The cytokines inside the exosomes could promote differentiation of T cells through rapid rearrangement of the T cell surface especially membrane receptors sensing the stimulation of cytokines. IL-4 blocking antibodies inhibit the differentiation of T cells might through direct interaction with IL-4 rapidly released from exosomes or targeted cells. Several studies reported that exosomes derived from patients could promote proliferation or differentiation of T cells (37, 42, 43). However, the detailed mechanism of exosome-mediated differentiation of T cells is awaiting further investigation.

The CFSE assay results showed that treatment with exosomes harvested from septic mice (Exo-24) but not those from control mice (Exo-0) had significant effects on the proliferation of lymphocytes activated by ConA (Figure 4A). Further studies with TLR4 knockout mice demonstrated that the proliferation of T cells was TLR4 receptor-independent (Supplementary Figure 4A). To eliminate the interference effect of endotoxin contamination in exosome preparations, we used PMB to neutralize LPS and found that the proliferation of T cells was not caused by endotoxin contamination (Supplementary Figure 4B).

Previous studies demonstrated that the cytokines/chemokines were involved in the proliferation of lymphocytes (44, 45). Our study found that IL-4, IL-10, IL-12, IL-17, IFN-γ, CCL5, CXCL9, and CXCL10 were significantly increased in the EXO-24h from septic mice (Figure 2). IL-2 had been reported with the function to promote T lymphocyte proliferation (44, 45), however, we failed to detect any increase of IL-2 in the exosomes from septic mice. Thus, we speculate that the high levels of IL-4, IFN-γ and CXCL9 associated with EXO-24h are the main causes promoting lymphocyte proliferation.

Migration of immune cells to infection sites is a key step to limit and kill pathogens in the development of infectious diseases. Microparticles (MPs) from mycobacteria-infected macrophages reportedly enhance the release of proinflammatory cytokines and chemokines and contribute to the disruption of respiratory epithelial cell integrity, providing a mechanism for cell recruitment to the site of *M. tuberculosis* infection in the lung (46, 47). In this study, we demonstrated that the exosomes harvested from septic mice failed to directly recruit lymphocytes but enhanced the migration of lymphocytes by direct physical interaction, which was supported by analysis of the air pouch model *in vivo*. However, the mechanisms underlying lymphocyte chemotaxis induced by preincubation with exosomes isolated from septic mice require further investigation.

Recent studies have shown that exosomes are involved in the inflammatory and immune responses and widely used to produce effective vaccines or treat immune disorders. Del Cacho et al. demonstrated that preimmunization of chickens with serum exosomes from *Eimeria tenella*-infected chickens induced protective immunity against experimental *E. tenella* (48). Yu et al. (40) reported that treatment with mTGF-β1-EXOs decreased the severity of experimental autoimmune encephalomyelitis (EAE) in BALB/c mice. In this study, we observed that pretreatment with exosomes from septic mice did exert significant biological effects. We found that administration of exosomes from septic mice inhibited the production of TNF-α and IL-10 in the serum, alleviated tissue injuries in the lung and liver and prolonged the survival times of mice subjected to CLP modeling. TNF-α is a vital proinflammatory cytokine, and IL-10 is a vital anti-inflammatory factor; both cytokines are involved in the development of sepsis. Suppression of TNF-α and IL-10 production provided a reasonable explanation for the remissive tissue injury and prolonged survival rates of mice.

It had been reported that exosomes could be released from platelets, leukocytes, endothelial cells, and other cells under physiological or pathological conditions (49, 50). In this study, we speculate that the origin of exosomes in the animals with sepsis is a mixture from different cells, including neutrophils, macrophages, lymphocytes, NK cells, endothelial cells, and platelets. However, the origin of cytokine-laden exosomes in serum remains to be fully elucidated.

In summary, this study demonstrated that most cytokines/chemokines in septic mouse serum exist in two forms, i.e., the soluble free form and the insoluble exosomal form. For the first time, we herein revealed that the exosomes from septic mice were immunoreactive and had the capability to enhance Th1/Th2 differentiation, promote T cell proliferation and augment T lymphocyte migration. Preadministration of exosomes from septic mice not only suppressed cytokine production and alleviated tissue injury but also prolonged the survival of CLP mice. Characterizing the role of exosomes in sepsis would strengthen our understanding of the mechanisms underlying sepsis development and provide potential targets for therapeutic applications (51).

## Data Availability

The raw data supporting the conclusions of this manuscript will be made available by the authors, without undue reservation, to any qualified researcher.

## Ethics Statement

All applicable international, national and/or institutional guidelines for the care and use of animals were followed.

## Author Contributions

YJ conceived and designed the work. KG, JJ, CH, JL, and LL performed the experiment. HL, CH, and YH discussed, wrote, and edited the manuscript. YJ revised the manuscript. All authors read and approved the final manuscript.

### Conflict of Interest Statement

The authors declare that the research was conducted in the absence of any commercial or financial relationships that could be construed as a potential conflict of interest.
