# Supplementary material for: Exosomes Derived From Septic Mouse Serum Modulate Immune Responses via Exosome-Associated Cytokines
Source: Front Immunol. 2019 Jul 12;10:1560. doi: 10.3389/fimmu.2019.01560 (PMC6640201; doi:10.3389/fimmu.2019.01560)
Supplement: Supplementary file 1 [file Data_Sheet_1.pdf]

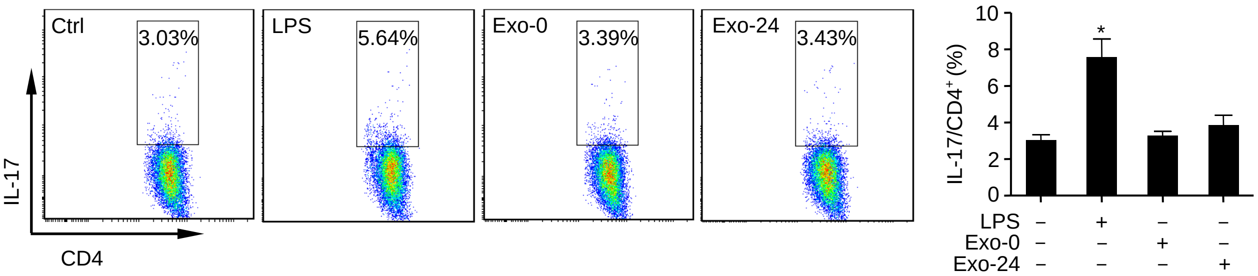

Supplementary Fig. 1 Exosomes from septic mice failed to induce Th17 differentiation.

Lymphocytes were purified from the spleens of C57BL/6 mice and incubated with LPS (1 μg/ml) or exosomes (Exo-0 or Exo-24, 10 μg/ml) for 72 h. Flow cytometry was performed to determine the percentage of Th17 cells by calculating the ratio of IL-17-expressing cells to $CD4^+$ T cells. Lymphocytes stimulated with LPS for 72 h (LPS group) were used as the positive control. Data are presented as the mean±SD (*n*=4), * *P*<0.05 versus the control group.

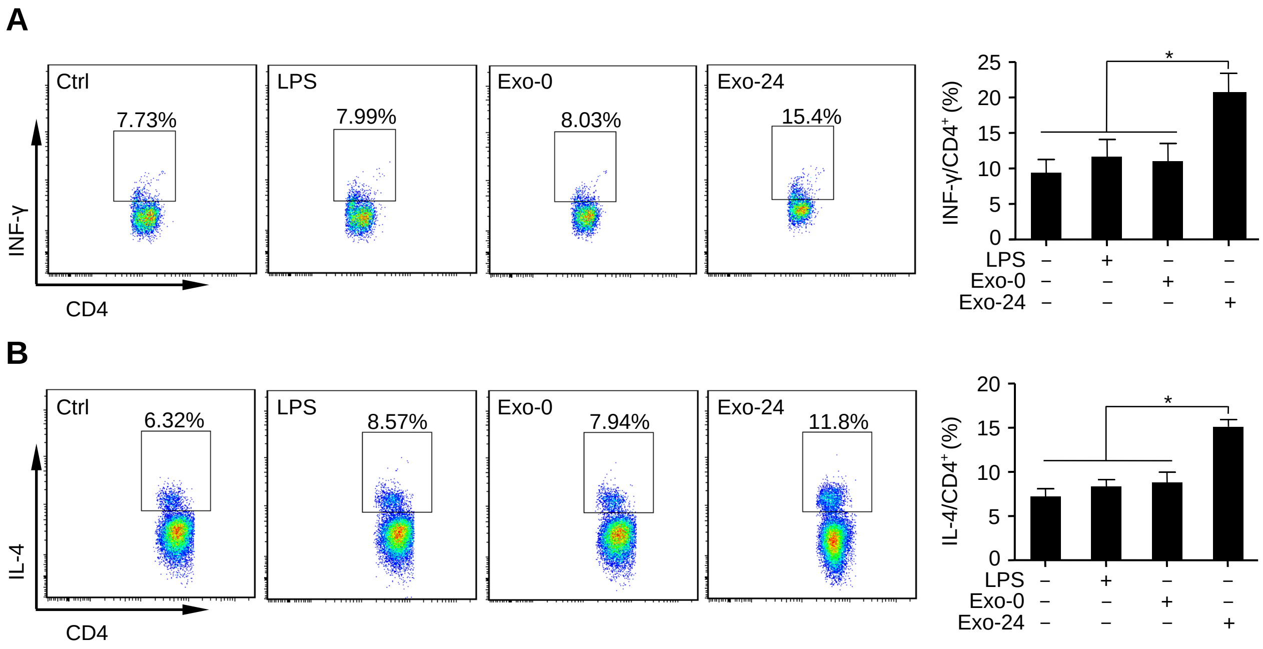

Supplementary Fig. 2 Exosomes from septic mice augmented Th1 and Th2 differentiation in a TLR4-independent manner.

(A) Th1 differentiation was induced by exosomes from septic mice in TLR4-deficient lymphocytes. Lymphocytes were purified from the spleens of $TLR4^{-/-}$ C57BL/6 mice and incubated with LPS (1 μg/ml) or exosomes (Exo-0 or Exo-24, 10 μg/ml) for 72 h. Flow cytometry was performed to determine the percentage of Th1 cells by calculating the ratio of IFN-γ-expressing cells to $CD4^+$ T cells. Data are presented as the mean±SD (*n*=4), * P<0.05 versus the control, LPS and Exo-0 groups. (B) The Th2 response was enhanced by exosomes from septic mice in TLR4-deficient lymphocytes. Splenic lymphocytes were acquired and incubated with LPS or exosomes (Exo-0 or Exo-24) for 72 h. The percentage of Th2 cells was calculated by the ratio of IL-4-expressing cells to $CD4^+$ T cells as determined by flow cytometry. Data are presented as the mean±SD (*n*=4), * P<0.05 versus the control, LPS and Exo-0 groups.

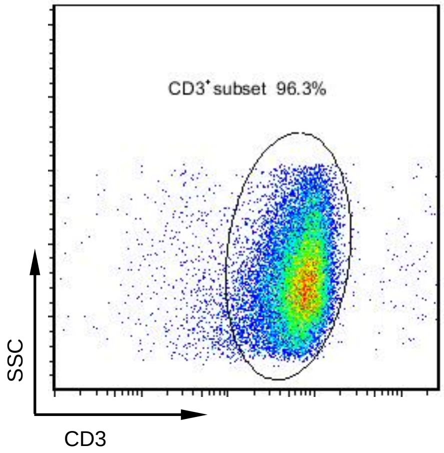

Supplementary Fig. 3 The purity of $CD3^+$ T cells extracted from splenic lymphocytes. Splenic lymphocytes were collected from C57BL/6 mice for the purification of T cells using a Pan T cell isolation kit as described in the Method. T cell purity was determined by FACS analysis using the anti-CD3-APC mAb.

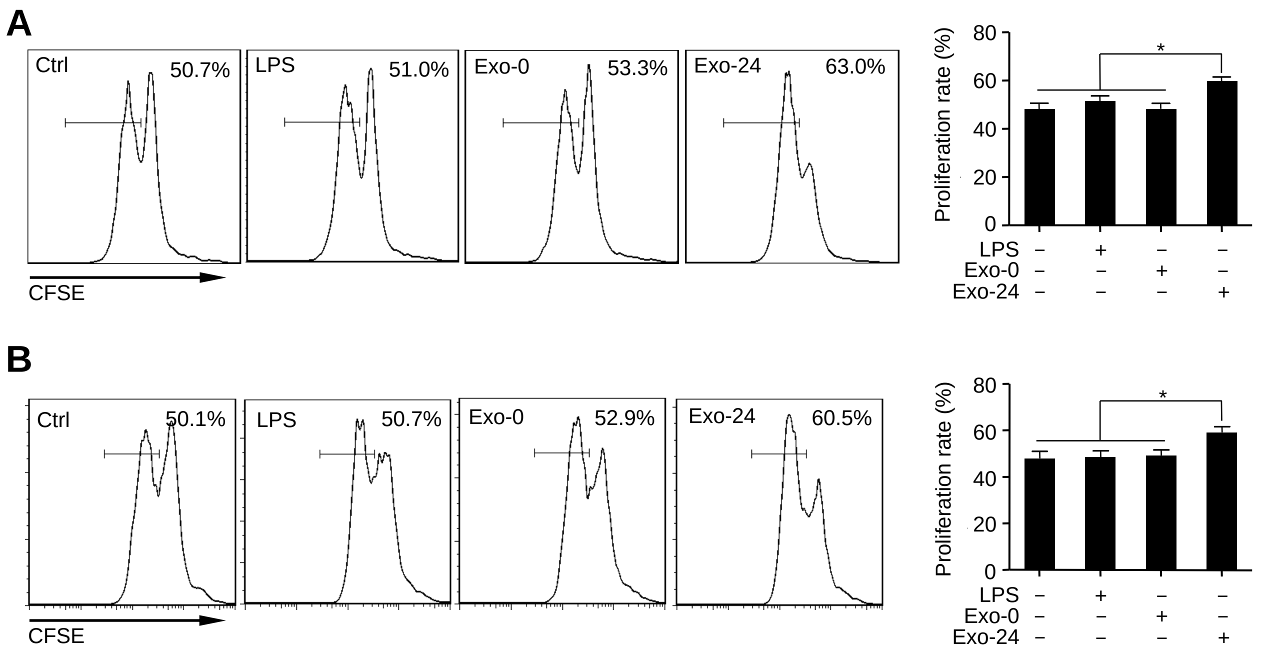

Supplementary Fig. 4 Exosomes from septic mice augmented lymphocyte proliferation in an endotoxin-independent manner.

$CFSE_{low}$ cells by FACS. Data are presented as the mean±SD (*n*=4). * *P*<0.05 versus the control, LPS and Exo-0 groups. (B) PMB failed to restrain the proliferation of lymphocytes induced by exosomes from septic mice. After preincubation with PMB (10 μg/mL) at room temperature for 1 h, exosomes (Exo-0 or Exo-24, 10 μg/ml) or LPS (1 μg/ml) were used to stimulate the splenic lymphocytes with CFSE for 72 h. Cell proliferation was quantitated as the percentage of $CFSE_{low}$ cells by FACS. Data are presented as the mean±SD (*n*=4). * *P*<0.05 versus the control, LPS and Exo-0 groups.

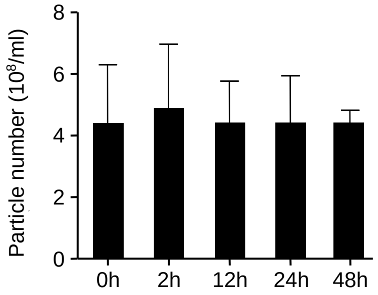

Supplementary Fig. 5 The number of exosome particles in the serum of septic mice at different time points.
